# Crystal structure of (1,3-di-*tert*-butyl-η^5^-cyclo­penta­dien­yl)tri­methyl­hafnium(IV)

**DOI:** 10.1107/S205698901500585X

**Published:** 2015-04-02

**Authors:** Adrián Pérez-Redondo, Víctor Varela-Izquierdo, Carlos Yélamos

**Affiliations:** a Departamento de Química Orgánica y Química Inorgánica, Universidad de Alcalá, Campus Universitario, ES 28871 Alcalá de Henares (Madrid), Spain

**Keywords:** crystal structure, hafnium, cyclo­penta­dienyl ligand, organometallic compound

## Abstract

The mol­ecule of the title organometallic hafnium(IV) com­pound, [Hf(CH_3_)_3_(C_13_H_21_)] or [HfMe_3_(η^5^-C_5_H_3_-1,3-^*t*^Bu_2_)], adopts the classical three-legged piano-stool geometry for mono­cyclo­penta­dienylhafnium(IV) derivatives with the three methyl groups bonded to the Hf(IV) atom at the legs. The C atoms of the two *tert*-butyl group bonded to the cyclo­penta­dienyl (Cp) ring are 0.132 (5) and 0.154 (6) Å above the Cp least-squares plane. There are no significant inter­molecular inter­actions present between the mol­ecules in the crystal structure.

## Related literature   

The synthesis of the compound was described by Cuenca *et al.* (1996 ▸). For the structures of related Hf^IV^ derivatives and a comparison of Hf—C bond lengths, see: Itagaki *et al.* (2009 ▸); Schäfer *et al.* (2013 ▸); Shah *et al.* (1996 ▸); Swenson *et al.* (2000 ▸).
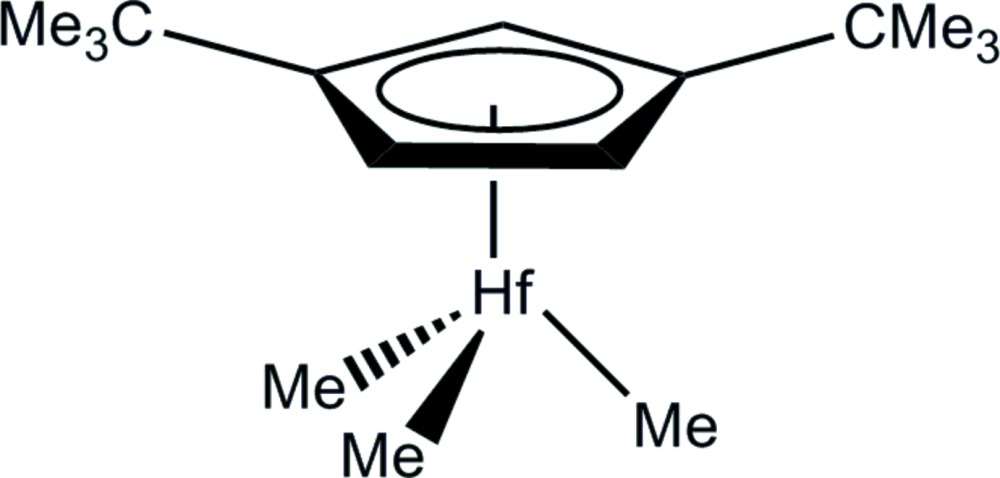


## Experimental   

### Crystal data  

[Hf(CH_3_)_3_(C_13_H_21_)]
*M* *_r_* = 400.89
Monoclinic, 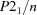

*a* = 13.238 (3) Å
*b* = 9.613 (2) Å
*c* = 14.486 (3) Å
β = 109.63 (2)°
*V* = 1736.3 (7) Å^3^
*Z* = 4
Mo *K*α radiation
μ = 5.99 mm^−1^
*T* = 200 K
0.42 × 0.14 × 0.11 mm

### Data collection  

Nonius KappaCCD diffractometer
Absorption correction: multi-scan (Blessing, 1995 ▸) *T*_min_ = 0.297, *T*_max_ = 0.531
29484 measured reflections
3136 independent reflections
2471 reflections with *I* > 2σ(*I*)
*R*_int_ = 0.100

### Refinement  

*R*[*F* ^2^ > 2σ(*F* ^2^)] = 0.032
*wR*(*F* ^2^) = 0.073
*S* = 1.12
3136 reflections
164 parameters
H-atom parameters constrained
Δρ_max_ = 1.42 e Å^−3^
Δρ_min_ = −1.35 e Å^−3^

### 

Data collection: *COLLECT* (Nonius, 1998 ▸); cell refinement: *DIRAX* (Duisenberg *et al.*, 2000 ▸); data reduction: *EVALCCD* (Duisenberg *et al.*, 2003 ▸); program(s) used to solve structure: *SHELXS97* (Sheldrick, 2008 ▸); program(s) used to refine structure: *SHELXL2014*/7 (Sheldrick, 2015 ▸); molecular graphics: *ORTEP-3 for Windows* (Farrugia, 2012 ▸); software used to prepare material for publication: *WinGX* (Farrugia, 2012 ▸) and *publCIF* (Westrip, 2010 ▸).

## Supplementary Material

Crystal structure: contains datablock(s) I. DOI: 10.1107/S205698901500585X/wm5139sup1.cif

Structure factors: contains datablock(s) I. DOI: 10.1107/S205698901500585X/wm5139Isup2.hkl

Supporting information file. DOI: 10.1107/S205698901500585X/wm5139Isup3.mol

**Fig. 1.**
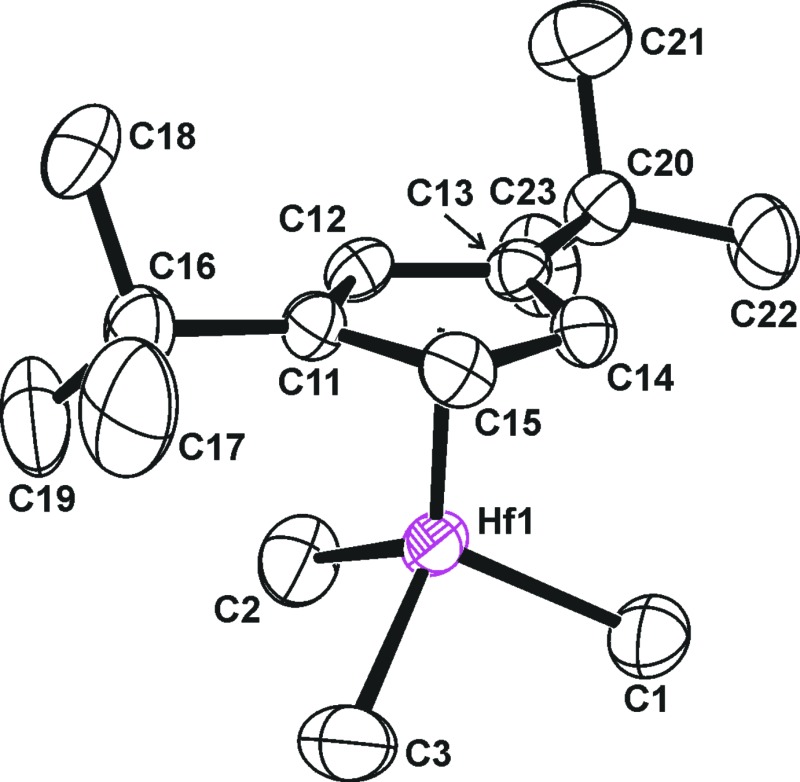
The molecular structure of [Hf(η5-1,3-tBu2C5H3)Me3] with displacement ellipsoids drawn at the 50% probability level. Hydrogen atoms are omitted for clarity.

5 t 2 5 3 3 . DOI: 10.1107/S205698901500585X/wm5139fig1.tif
The mol­ecular structure of [Hf(η^5^-1,3-^*t*^Bu_2_C_5_H_3_)Me_3_] with displacement ellipsoids drawn at the 50% probability level. Hydrogen atoms are omitted for clarity.

CCDC reference: 1055619

Additional supporting information:  crystallographic information; 3D view; checkCIF report

## Figures and Tables

**Table:** 

C1Hf1	2.198(6)
C2Hf1	2.211(6)
C3Hf1	2.213(6)
C11Hf1	2.519(5)
C12Hf1	2.500(4)
C13Hf1	2.524(4)
C14Hf1	2.484(5)
C15Hf1	2.468(5)

**Table:** 

C1Hf1C2	103.7(2)
C1Hf1C3	99.7(2)
C2Hf1C3	102.4(3)

## supplementary crystallographic information

### S1. Synthesis and crystallization

The title compound was synthesized according to a literature procedure (Cuenca *et al.*, 1996). Crystals were obtained from the resultant oil by removing the volatile components of a *n*-hexane solution.

### S2. Refinement

H atoms attached to *sp*^2^ C-atoms were placed geometrically, with C—H = 0.95 Å, and with *U*_iso_(H) = 1.2*U*_eq_(C). Methyl H-atoms were refined using a rotating-group model, with C—H = 0.98 Å, and with *U*_iso_(H) = 1.5*U*_eq_(C).

### Crystal data

**Table:**

[Hf(CH_3_)_3_(C_13_H_21_)]	*F*(000) = 792
*M**_r_* = 400.89	*D*_x_ = 1.534 Mg m^−^^3^
Monoclinic, *P*2_1_/*n*	Mo *K*α radiation, λ = 0.71073 Å
*a* = 13.238 (3) Å	Cell parameters from 117 reflections
*b* = 9.613 (2) Å	θ = 3–21°
*c* = 14.486 (3) Å	µ = 5.99 mm^−^^1^
β = 109.63 (2)°	*T* = 200 K
*V* = 1736.3 (7) Å^3^	Prism, colourless
*Z* = 4	0.42 × 0.14 × 0.11 mm

### Data collection

**Table:**

Nonius KappaCCD diffractometer	3136 independent reflections
Radiation source: Enraf Nonius FR590	2471 reflections with *I* > 2σ(*I*)
Horizonally mounted graphite crystal monochromator	*R*_int_ = 0.100
Detector resolution: 9 pixels mm^-1^	θ_max_ = 25.2°, θ_min_ = 3.3°
CCD scans	*h* = −15→15
Absorption correction: multi-scan (Blessing, 1995)	*k* = −11→10
*T*_min_ = 0.297, *T*_max_ = 0.531	*l* = −17→16
29484 measured reflections	

### Refinement

**Table:**

Refinement on *F*^2^	Secondary atom site location: structure-invariant direct methods
Least-squares matrix: full	Hydrogen site location: inferred from neighbouring sites
*R*[*F*^2^ > 2σ(*F*^2^)] = 0.032	H-atom parameters constrained
*wR*(*F*^2^) = 0.073	*w* = 1/[σ^2^(*F*_o_^2^) + (0.027*P*)^2^ + 1.470*P*] where *P* = (*F*_o_^2^ + 2*F*_c_^2^)/3
*S* = 1.12	(Δ/σ)_max_ < 0.001
3136 reflections	Δρ_max_ = 1.42 e Å^−^^3^
164 parameters	Δρ_min_ = −1.35 e Å^−^^3^
0 restraints	Extinction correction: *SHELXL2014*/7 (Sheldrick, 2015)
0 constraints	Extinction coefficient: 0.0113 (4)
Primary atom site location: structure-invariant direct methods	

### Special details

**Table:**

Geometry. All e.s.d.'s (except the e.s.d. in the dihedral angle between two l.s. planes) are estimated using the full covariance matrix. The cell e.s.d.'s are taken into account individually in the estimation of e.s.d.'s in distances, angles and torsion angles; correlations between e.s.d.'s in cell parameters are only used when they are defined by crystal symmetry. An approximate (isotropic) treatment of cell e.s.d.'s is used for estimating e.s.d.'s involving l.s. planes.

### Fractional atomic coordinates and isotropic or equivalent isotropic displacement parameters (Å^2^)

**Table:**

	*x*	*y*	*z*	*U*_iso_*/*U*_eq_	
C1	0.0972 (5)	−0.0651 (7)	0.2994 (4)	0.0639 (18)	
H1A	0.0395	−0.1199	0.3098	0.096*	
H1B	0.1367	−0.1231	0.2675	0.096*	
H1C	0.0664	0.015	0.2576	0.096*	
C2	0.3457 (5)	0.0906 (7)	0.4060 (5)	0.0643 (18)	
H2A	0.4003	0.0178	0.4168	0.096*	
H2B	0.376	0.1704	0.4483	0.096*	
H2C	0.3221	0.1199	0.3372	0.096*	
C3	0.1226 (5)	0.2003 (6)	0.4579 (5)	0.069 (2)	
H3A	0.0907	0.2447	0.3937	0.103*	
H3B	0.1737	0.2644	0.5024	0.103*	
H3C	0.066	0.177	0.4848	0.103*	
C11	0.2768 (4)	−0.0503 (5)	0.6213 (4)	0.0337 (11)	
C12	0.3349 (4)	−0.1313 (5)	0.5761 (3)	0.0338 (11)	
H12	0.4093	−0.1215	0.5863	0.041*	
C13	0.2663 (4)	−0.2296 (5)	0.5131 (3)	0.0326 (11)	
C14	0.1638 (4)	−0.2058 (5)	0.5167 (4)	0.0347 (11)	
H14	0.1007	−0.2555	0.4809	0.042*	
C15	0.1698 (4)	−0.0956 (5)	0.5824 (4)	0.0361 (12)	
H15	0.1111	−0.058	0.5979	0.043*	
C16	0.3228 (4)	0.0569 (6)	0.7029 (4)	0.0464 (14)	
C17	0.2339 (6)	0.1215 (8)	0.7320 (5)	0.085 (2)	
H17A	0.1948	0.0483	0.753	0.127*	
H17B	0.1845	0.1712	0.6758	0.127*	
H17C	0.2646	0.1869	0.786	0.127*	
C18	0.3969 (6)	−0.0205 (6)	0.7922 (5)	0.076 (2)	
H18A	0.4239	0.0443	0.8472	0.113*	
H18B	0.4574	−0.0601	0.7764	0.113*	
H18C	0.357	−0.0955	0.8102	0.113*	
C19	0.3869 (6)	0.1672 (6)	0.6724 (5)	0.077 (2)	
H19A	0.3404	0.2161	0.6144	0.116*	
H19B	0.4462	0.1232	0.6571	0.116*	
H19C	0.4158	0.2338	0.7261	0.116*	
C20	0.3013 (4)	−0.3463 (5)	0.4585 (4)	0.0425 (13)	
C21	0.3666 (6)	−0.4500 (6)	0.5375 (5)	0.071 (2)	
H21A	0.4269	−0.4014	0.585	0.106*	
H21B	0.3938	−0.5248	0.5064	0.106*	
H21C	0.3204	−0.4896	0.5714	0.106*	
C22	0.2040 (5)	−0.4224 (6)	0.3889 (5)	0.0629 (18)	
H22A	0.1602	−0.4593	0.426	0.094*	
H22B	0.2283	−0.4992	0.3571	0.094*	
H22C	0.1611	−0.3574	0.3389	0.094*	
C23	0.3706 (5)	−0.2918 (7)	0.4017 (5)	0.0697 (19)	
H23A	0.3286	−0.2277	0.3507	0.105*	
H23B	0.3953	−0.3699	0.3714	0.105*	
H23C	0.4327	−0.2427	0.4465	0.105*	
Hf1	0.20704 (2)	0.00819 (2)	0.44138 (2)	0.03781 (13)	

### Atomic displacement parameters (Å^2^)

**Table:**

	*U*^11^	*U*^22^	*U*^33^	*U*^12^	*U*^13^	*U*^23^
C1	0.065 (4)	0.068 (4)	0.049 (4)	−0.013 (3)	0.007 (3)	0.015 (3)
C2	0.064 (4)	0.077 (4)	0.054 (4)	−0.018 (3)	0.022 (3)	0.006 (3)
C3	0.057 (4)	0.051 (4)	0.088 (5)	0.007 (3)	0.011 (4)	0.018 (3)
C11	0.037 (3)	0.034 (2)	0.028 (3)	−0.002 (2)	0.008 (2)	−0.002 (2)
C12	0.026 (2)	0.042 (3)	0.032 (3)	0.003 (2)	0.008 (2)	0.003 (2)
C13	0.036 (3)	0.034 (3)	0.028 (3)	0.003 (2)	0.009 (2)	0.000 (2)
C14	0.031 (3)	0.034 (3)	0.040 (3)	−0.005 (2)	0.013 (2)	0.002 (2)
C15	0.033 (3)	0.038 (3)	0.043 (3)	0.000 (2)	0.019 (2)	0.002 (2)
C16	0.054 (3)	0.045 (3)	0.037 (3)	−0.006 (3)	0.012 (3)	−0.010 (2)
C17	0.090 (5)	0.093 (5)	0.075 (5)	0.001 (4)	0.031 (4)	−0.048 (4)
C18	0.089 (5)	0.067 (4)	0.042 (4)	−0.001 (3)	−0.016 (4)	−0.012 (3)
C19	0.104 (6)	0.070 (4)	0.055 (4)	−0.047 (4)	0.024 (4)	−0.018 (3)
C20	0.041 (3)	0.047 (3)	0.039 (3)	0.004 (2)	0.013 (2)	−0.007 (2)
C21	0.090 (5)	0.060 (4)	0.057 (4)	0.033 (4)	0.017 (4)	−0.008 (3)
C22	0.071 (4)	0.050 (4)	0.062 (4)	−0.007 (3)	0.016 (3)	−0.026 (3)
C23	0.072 (5)	0.071 (4)	0.079 (5)	−0.001 (3)	0.043 (4)	−0.027 (4)
Hf1	0.03159 (16)	0.04226 (18)	0.03505 (17)	−0.00414 (9)	0.00523 (10)	0.00804 (10)

### Geometric parameters (Å, º)

**Table:**

C1—Hf1	2.198 (6)	C15—H15	0.95
C1—H1A	0.98	C16—C17	1.511 (9)
C1—H1B	0.98	C16—C19	1.513 (8)
C1—H1C	0.98	C16—C18	1.529 (8)
C2—Hf1	2.211 (6)	C17—H17A	0.98
C2—H2A	0.98	C17—H17B	0.98
C2—H2B	0.98	C17—H17C	0.98
C2—H2C	0.98	C18—H18A	0.98
C3—Hf1	2.213 (6)	C18—H18B	0.98
C3—H3A	0.98	C18—H18C	0.98
C3—H3B	0.98	C19—H19A	0.98
C3—H3C	0.98	C19—H19B	0.98
C11—C12	1.402 (7)	C19—H19C	0.98
C11—C15	1.406 (6)	C20—C23	1.517 (8)
C11—C16	1.531 (7)	C20—C22	1.530 (7)
C11—Hf1	2.519 (5)	C20—C21	1.545 (7)
C12—C13	1.412 (6)	C21—H21A	0.98
C12—Hf1	2.500 (4)	C21—H21B	0.98
C12—H12	0.95	C21—H21C	0.98
C13—C14	1.395 (6)	C22—H22A	0.98
C13—C20	1.531 (7)	C22—H22B	0.98
C13—Hf1	2.524 (4)	C22—H22C	0.98
C14—C15	1.409 (7)	C23—H23A	0.98
C14—Hf1	2.484 (5)	C23—H23B	0.98
C14—H14	0.95	C23—H23C	0.98
C15—Hf1	2.468 (5)		
			
Hf1—C1—H1A	109.5	H18A—C18—H18B	109.5
Hf1—C1—H1B	109.5	C16—C18—H18C	109.5
H1A—C1—H1B	109.5	H18A—C18—H18C	109.5
Hf1—C1—H1C	109.5	H18B—C18—H18C	109.5
H1A—C1—H1C	109.5	C16—C19—H19A	109.5
H1B—C1—H1C	109.5	C16—C19—H19B	109.5
Hf1—C2—H2A	109.5	H19A—C19—H19B	109.5
Hf1—C2—H2B	109.5	C16—C19—H19C	109.5
H2A—C2—H2B	109.5	H19A—C19—H19C	109.5
Hf1—C2—H2C	109.5	H19B—C19—H19C	109.5
H2A—C2—H2C	109.5	C23—C20—C22	109.6 (5)
H2B—C2—H2C	109.5	C23—C20—C13	111.8 (4)
Hf1—C3—H3A	109.5	C22—C20—C13	110.9 (4)
Hf1—C3—H3B	109.5	C23—C20—C21	109.6 (5)
H3A—C3—H3B	109.5	C22—C20—C21	108.5 (5)
Hf1—C3—H3C	109.5	C13—C20—C21	106.3 (4)
H3A—C3—H3C	109.5	C20—C21—H21A	109.5
H3B—C3—H3C	109.5	C20—C21—H21B	109.5
C12—C11—C15	106.1 (4)	H21A—C21—H21B	109.5
C12—C11—C16	126.5 (4)	C20—C21—H21C	109.5
C15—C11—C16	127.2 (5)	H21A—C21—H21C	109.5
C12—C11—Hf1	73.0 (3)	H21B—C21—H21C	109.5
C15—C11—Hf1	71.6 (3)	C20—C22—H22A	109.5
C16—C11—Hf1	124.1 (4)	C20—C22—H22B	109.5
C11—C12—C13	109.8 (4)	H22A—C22—H22B	109.5
C11—C12—Hf1	74.5 (3)	C20—C22—H22C	109.5
C13—C12—Hf1	74.6 (3)	H22A—C22—H22C	109.5
C11—C12—H12	125.1	H22B—C22—H22C	109.5
C13—C12—H12	125.1	C20—C23—H23A	109.5
Hf1—C12—H12	117.6	C20—C23—H23B	109.5
C14—C13—C12	106.8 (4)	H23A—C23—H23B	109.5
C14—C13—C20	127.3 (4)	C20—C23—H23C	109.5
C12—C13—C20	125.7 (4)	H23A—C23—H23C	109.5
C14—C13—Hf1	72.3 (3)	H23B—C23—H23C	109.5
C12—C13—Hf1	72.7 (3)	C1—Hf1—C2	103.7 (2)
C20—C13—Hf1	123.8 (3)	C1—Hf1—C3	99.7 (2)
C13—C14—C15	108.3 (4)	C2—Hf1—C3	102.4 (3)
C13—C14—Hf1	75.4 (3)	C1—Hf1—C15	113.1 (2)
C15—C14—Hf1	72.8 (3)	C2—Hf1—C15	138.5 (2)
C13—C14—H14	125.9	C3—Hf1—C15	90.0 (2)
C15—C14—H14	125.9	C1—Hf1—C14	88.1 (2)
Hf1—C14—H14	117.8	C2—Hf1—C14	136.9 (2)
C11—C15—C14	108.9 (4)	C3—Hf1—C14	116.4 (2)
C11—C15—Hf1	75.6 (3)	C15—Hf1—C14	33.1 (2)
C14—C15—Hf1	74.1 (3)	C1—Hf1—C12	128.3 (2)
C11—C15—H15	125.5	C2—Hf1—C12	88.6 (2)
C14—C15—H15	125.5	C3—Hf1—C12	126.7 (2)
Hf1—C15—H15	116.7	C15—Hf1—C12	53.7 (2)
C17—C16—C19	111.0 (6)	C14—Hf1—C12	53.8 (2)
C17—C16—C18	107.6 (6)	C1—Hf1—C11	142.6 (2)
C19—C16—C18	108.9 (5)	C2—Hf1—C11	106.0 (2)
C17—C16—C11	110.3 (5)	C3—Hf1—C11	95.6 (2)
C19—C16—C11	111.3 (5)	C15—Hf1—C11	32.7 (2)
C18—C16—C11	107.5 (5)	C14—Hf1—C11	54.5 (2)
C16—C17—H17A	109.5	C12—Hf1—C11	32.5 (2)
C16—C17—H17B	109.5	C1—Hf1—C13	96.4 (2)
H17A—C17—H17B	109.5	C2—Hf1—C13	104.6 (2)
C16—C17—H17C	109.5	C3—Hf1—C13	144.1 (2)
H17A—C17—H17C	109.5	C15—Hf1—C13	54.1 (2)
H17B—C17—H17C	109.5	C14—Hf1—C13	32.3 (2)
C16—C18—H18A	109.5	C12—Hf1—C13	32.6 (2)
C16—C18—H18B	109.5	C11—Hf1—C13	54.3 (2)
			
C15—C11—C12—C13	−2.5 (6)	Hf1—C14—C15—C11	−68.3 (3)
C16—C11—C12—C13	172.8 (5)	C13—C14—C15—Hf1	67.7 (3)
Hf1—C11—C12—C13	−66.9 (3)	C12—C11—C16—C17	−179.8 (6)
C15—C11—C12—Hf1	64.5 (3)	C15—C11—C16—C17	−5.4 (8)
C16—C11—C12—Hf1	−120.2 (5)	Hf1—C11—C16—C17	86.6 (6)
C11—C12—C13—C14	2.1 (5)	C12—C11—C16—C19	56.5 (8)
Hf1—C12—C13—C14	−64.7 (3)	C15—C11—C16—C19	−129.2 (6)
C11—C12—C13—C20	−173.5 (5)	Hf1—C11—C16—C19	−37.1 (6)
Hf1—C12—C13—C20	119.6 (5)	C12—C11—C16—C18	−62.7 (7)
C11—C12—C13—Hf1	66.8 (3)	C15—C11—C16—C18	111.6 (6)
C12—C13—C14—C15	−1.0 (5)	Hf1—C11—C16—C18	−156.3 (5)
C20—C13—C14—C15	174.6 (5)	C14—C13—C20—C23	134.0 (5)
Hf1—C13—C14—C15	−66.0 (3)	C12—C13—C20—C23	−51.3 (7)
C12—C13—C14—Hf1	65.0 (3)	Hf1—C13—C20—C23	41.1 (6)
C20—C13—C14—Hf1	−119.4 (5)	C14—C13—C20—C22	11.3 (7)
C12—C11—C15—C14	1.8 (6)	C12—C13—C20—C22	−174.0 (5)
C16—C11—C15—C14	−173.4 (5)	Hf1—C13—C20—C22	−81.6 (5)
Hf1—C11—C15—C14	67.3 (3)	C14—C13—C20—C21	−106.5 (6)
C12—C11—C15—Hf1	−65.4 (3)	C12—C13—C20—C21	68.2 (7)
C16—C11—C15—Hf1	119.3 (5)	Hf1—C13—C20—C21	160.6 (4)
C13—C14—C15—C11	−0.6 (6)		
