# Supplementary figures and images for: Crystal structure of (1,3-di-tert-butyl-η5-cyclo­penta­dien­yl)tri­methyl­hafnium(IV)
Source: Acta Crystallogr E Crystallogr Commun. 2015 Apr 2;71(Pt 5):m100–1. doi: 10.1107/S205698901500585X (PMC4420044; doi:10.1107/S205698901500585X)

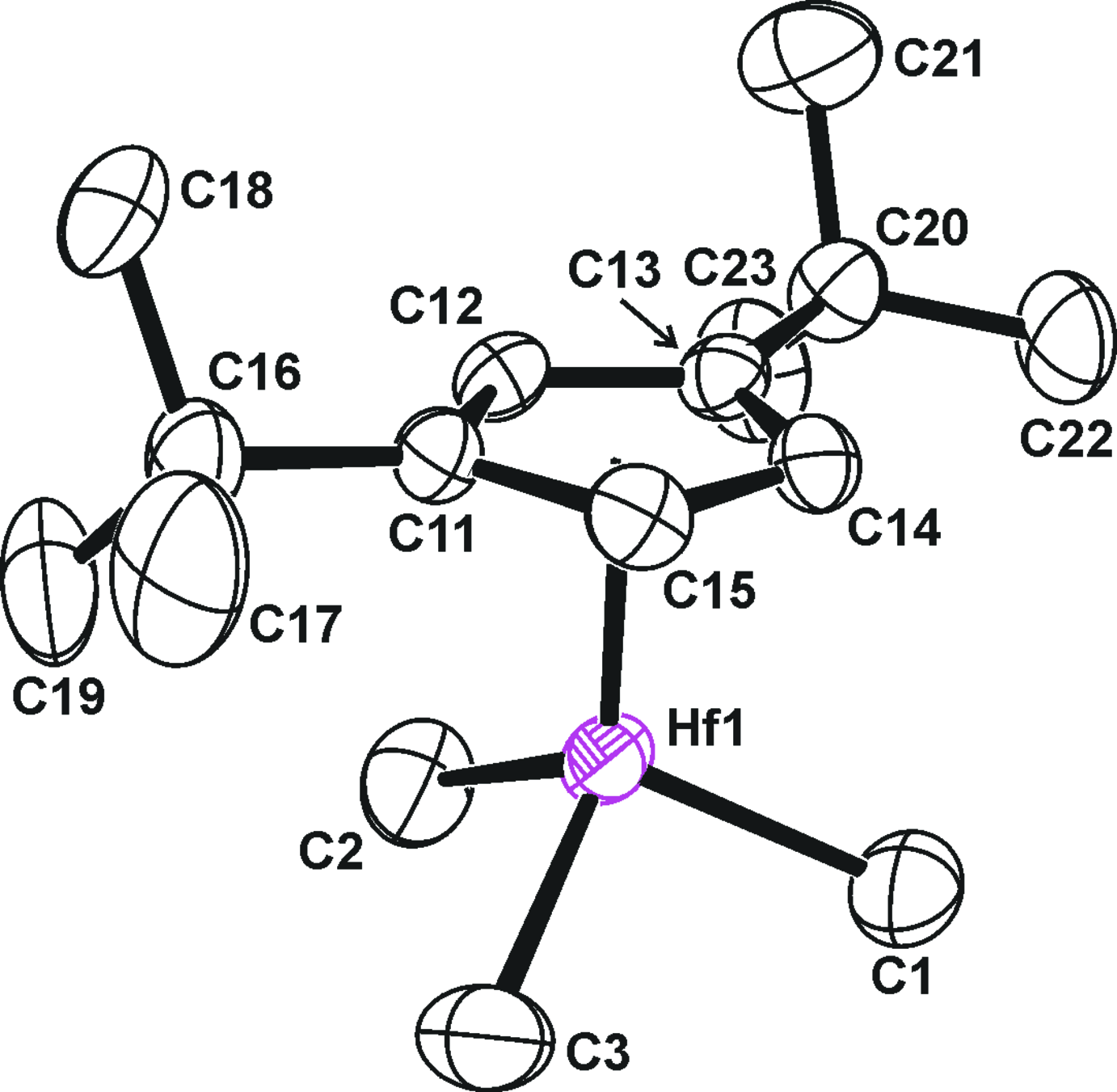

Supplement: Supplementary file 4 [file e-71-0m100-fig1.tif]
